# Aerosol and Surface Distribution of Severe Acute Respiratory Syndrome Coronavirus 2 in Hospital Wards, Wuhan, China, 2020

**DOI:** 10.3201/eid2607.200885

**Published:** 2020-07

**Authors:** Zhen-Dong Guo, Zhong-Yi Wang, Shou-Feng Zhang, Xiao Li, Lin Li, Chao Li, Yan Cui, Rui-Bin Fu, Yun-Zhu Dong, Xiang-Yang Chi, Meng-Yao Zhang, Kun Liu, Cheng Cao, Bin Liu, Ke Zhang, Yu-Wei Gao, Bing Lu, Wei Chen

**Affiliations:** Academy of Military Medical Sciences, Beijing, China (Z.-D. Guo, Z.-Y. Wang, S.-F. Zhang, X. Li, L. Li, Y.-Z. Dong, X.-Y. Chi, M.-Y. Zhang, C. Cao, K. Zhang, Y.-W. Gao, B. Lu, W. Chen);; Institute of Medical Support Technology, Institute of Systems Engineering, Academy of Military Sciences, Tianjin, China (C. Li);; Wuhan Huoshenshan Hospital, Wuhan, China (Y. Cui, R.-B. Fu, B. Liu);; Central Theater General Hospital, Wuhan (K. Liu)

**Keywords:** COVID-19, 2019 novel coronavirus disease, coronavirus disease, SARS-CoV-2, severe acute respiratory syndrome coronavirus 2, viruses, respiratory infections, zoonoses, exposure risk, medical staff protection, hospital-associated infection, aerosol, Wuhan, China

## Abstract

To determine distribution of severe acute respiratory syndrome coronavirus 2 in hospital wards in Wuhan, China, we tested air and surface samples. Contamination was greater in intensive care units than general wards. Virus was widely distributed on floors, computer mice, trash cans, and sickbed handrails and was detected in air ≈4 m from patients.

As of March 30, 2020, approximately 750,000 cases of coronavirus disease (COVID-19) had been reported globally since December 2019 (*1*), severely burdening the healthcare system (*2*). The extremely fast transmission capability of severe acute respiratory syndrome coronavirus 2 (SARS-CoV-2) has aroused concern about its various transmission routes.

The main transmission routes for SARS-CoV-2 are respiratory droplets and close contact (*3*). Knowing the extent of environmental contamination of SARS-CoV-2 in COVID-19 wards is critical for improving safety practices for medical staff and answering questions about SARS-CoV-2 transmission among the public. However, whether SARS-CoV-2 can be transmitted by aerosols remains controversial, and the exposure risk for close contacts has not been systematically evaluated. Researchers have detected SARS-CoV-2 on surfaces of objects in a symptomatic patient’s room and toilet area (*4*). However, that study was performed in a small sample from regions with few confirmed cases, which might not reflect real conditions in outbreak regions where hospitals are operating at full capacity. In this study, we tested surface and air samples from an intensive care unit (ICU) and a general COVID-19 ward (GW) at Huoshenshan Hospital in Wuhan, China (Figure 1).

**Figure 1:**
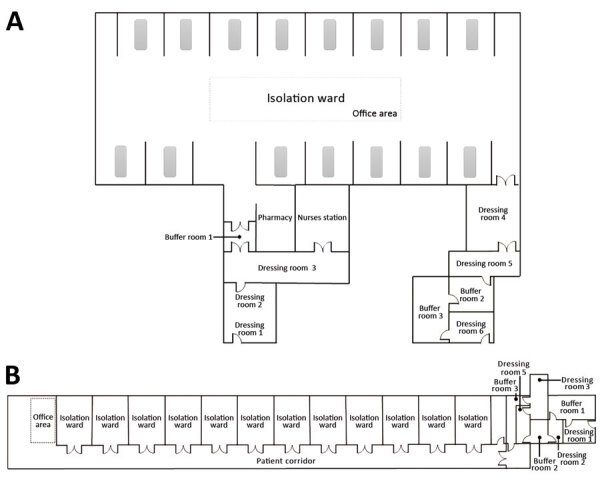
Layout of the intensive care unit (ICU) (A) and general ward (B) at Huoshenshan Hospital, Wuhan, China. For the ICU, the order of dressing is dressing room 1, dressing room 2, and dressing room 3; the order of undressing is dressing room 4, dressing room 5, and dressing room 6. The isolation ward of ICU is a large floor space with 15 cubicles (each with a patient bed) along the 2 opposite perimeters. Each cubicle is open to the central open area without any partition. For the general ward, the order of dressing is dressing room 1, dressing room 2, and dressing room 3; the order of undressing is dressing room 4, dressing room 5, and buffer room 1. The contaminated area of the general ward contains a patient corridor, and the 1-sided cubicles are all enclosed with door access to the corridor.

## The Study

From February 19 through March 2, 2020, we collected swab samples from potentially contaminated objects in the ICU and GW as described previously (*5*). The ICU housed 15 patients with severe disease and the GW housed 24 patients with milder disease. We also sampled indoor air and the air outlets to detect aerosol exposure. Air samples were collected by using a SASS 2300 Wetted Wall Cyclone Sampler (Research International, Inc., https://www.resrchintl.com) at 300 L/min for of 30 min. We used sterile premoistened swabs to sample the floors, computer mice, trash cans, sickbed handrails, patient masks, personal protective equipment, and air outlets. We tested air and surface samples for the open reading frame (ORF) *1ab* and nucleoprotein (N) genes of SARS-CoV-2 by quantitative real-time PCR. (Appendix).

Almost all positive results were concentrated in the contaminated areas (ICU 54/57, 94.7%; GW 9/9, 100%); the rate of positivity was much higher for the ICU (54/124, 43.5%) than for the GW (9/114, 7.9%) (Tables 1, 2). The rate of positivity was relatively high for floor swab samples (ICU 7/10, 70%; GW 2/13, 15.4%), perhaps because of gravity and air flow causing most virus droplets to float to the ground. In addition, as medical staff walk around the ward, the virus can be tracked all over the floor, as indicated by the 100% rate of positivity from the floor in the pharmacy, where there were no patients. Furthermore, half of the samples from the soles of the ICU medical staff shoes tested positive. Therefore, the soles of medical staff shoes might function as carriers. The 3 weak positive results from the floor of dressing room 4 might also arise from these carriers. We highly recommend that persons disinfect shoe soles before walking out of wards containing COVID-19 patients.

**Table 1:** Results of testing for SARS-CoV-2 in intensive care unit, Huoshenshan Hospital, Wuhan, China, 2020*

Area, sample	Intense positive/weak positive/negative†	Rate of positivity, %	Average virus concentration‡
Contaminated area			
Isolation wards			
Floor	6/1/3	70	6.6 × 10^4^
Computer mouse	4/2/2	75	2.8 × 10^4^
Trash can	0/3/2	60	3.4 × 10^4^
Sickbed handrail	2/4/8	42.9	4.3 × 10^4^
Patient mask	1/1/3	40	3.3 × 10^3^
Air outlet filter	4/4/4	66.7	1.5 × 10^5^
Indoor air near the air outlet (sampling site 1 in Figure 2, panel A)	2/3/9	35.7	3.8
Indoor air near the patients (sampling site 2 in Figure 2, panel A)	2/6/10	44.4	1.4
Indoor air near the doctors’ office area (sampling site 3 in Figure 2, panel A)	0/1/7	12.5	0.52
Pharmacy			
Floor	3/0/0	100	7.45 × 10^4^
Indoor air	0/0/5	0	ND
PPE			
Face shield of medical staff	0/0/6	0	ND
Sleeve cuff of medical staff	0/1/5	16.7	7.1 × 10^3^
Glove of medical staff	0/1/3	25	2.9 × 10^3^
Shoe sole of medical staff	3/0/3	50	3.2 × 10^4^
Subtotal	27/27/70	43.5	NA
Semicontaminated area			
Buffer room 1			
Floor	0/0/5	0	ND
Air outlet filter	0/0/3	0	ND
Indoor air	0/0/5	0	ND
Doorknob	0/0/3	0	ND
Dressing room 4			
Floor	0/3/5	37.5	3.8 × 10^3^
Air outlet filter	0/0/3	0	ND
Indoor air	0/0/5	0	ND
Doorknob	0/0/4	0	ND
Subtotal	0/3/33	8.3	NA
Clean area			
Dressing rooms 1, 2, and 3			
Doorknob	0/0/10	0	ND
Floor	0/0/12	0	ND
Indoor air	0/0/8	0	ND
Nurse station			
Doorknob	0/0/5	0	ND
Floor	0/0/5	0	ND
Indoor air	0/0/5	0	ND
Dressing rooms 5 and 6, buffer rooms 2 and 3			
Doorknob	0/0/12	0	ND
Floor	0/0/12	0	ND
Indoor air	0/0/12	0	ND
Subtotal			
Total	27/30/184	23.7	NA

*NA, not applicable; ND, not determined; PPE, personal protective equipment; SARS-CoV-2, severe acute respiratory syndrome coronavirus 2. †Intense positive indicates a positive result for both open reading frame *1ab* gene and nucleoprotein gene of SARS-CoV-2; weak positive indicates a positive result for only 1 of the genes. ‡The average virus concentration of indoor air expressed as copies/L and of swab samples, as copies/sample.

**Table 2:** Results of testing for SARS-CoV-2 in general ward, Huoshenshan Hospital, Wuhan, China, 2020*

Area, sample	Intense positive/weak positive/negative†	Rate of positivity, %	Average virus concentration‡
Contaminated area			
Isolation ward			
Floor	1/1/11	15.4	1.6 × 10^4^
Doorknob	0/1/11	8.3	6.5 × 10^2^
Air outlet	0/1/11	8.3	3.4 × 10^3^
Sickbed handrail	0/0/12	0	ND
Patient mask	1/1/8	20	9.2 × 10^3^
Indoor air (sampling site 1 in Figure 2, panel C)	0/2/9	18.2	0.68
Indoor air (sampling site 2 in Figure 2, panel C)	0/0/5	0	ND
Patient corridor			
Floor	0/0/10	0	ND
Computer mouse or keyboard	0/1/4	20	3.9 × 10^3^
Trash can	0/0/8	0	ND
Indoor air	0/0/4	0	ND
PPE			
Face shield of medical staff	0/0/3	0	ND
Sleeve cuff of medical staff	0/0/3	0	ND
Glove of medical staff	0/0/3	0	ND
Shoe sole of medical staff	0/0/3	0	ND
Subtotal	2/7/105	7.9	NA
Semicontaminated area			
Dressing Room 4			
Floor	0/0/5	0	ND
Indoor air	0/0/5	0	ND
Doorknob	0/0/3	0	ND
Buffer Room 3			
Floor	0/0/5	0	ND
Indoor air	0/0/3	0	ND
Doorknob	0/0/3	0	ND
Subtotal	0/0/24	0	NA
Clean area			
Dressing Rooms 1, 2, 3, and 5			
Doorknob	0/0/12	0	ND
Floor	0/0/12	0	ND
Indoor air	0/0/6	0	ND
Buffer rooms 1 and 2			
Doorknob	0/0/6	0	ND
Floor	0/0/6	0	ND
Indoor air	0/0/4	0	ND
Subtotal	0/0/46	0	NA
Total	2/7/175	4.9	NA

*NA, not applicable; ND, not determined; PPE, personal protective equipment; SARS-CoV-2, severe acute respiratory syndrome coronavirus 2. †Intense positive indicates a positive result for both open reading frame *1ab* gene and nucleoprotein gene of SARS-CoV-2; weak positive indicates a positive result for only 1 of the genes. ‡The average virus concentration of indoor air expressed as copies/L and of swab samples, as copies/sample.

The rate of positivity was also relatively high for the surface of the objects that were frequently touched by medical staff or patients (Tables 1, 2). The highest rates were for computer mice (ICU 6/8, 75%; GW 1/5, 20%), followed by trash cans (ICU 3/5, 60%; GW 0/8), sickbed handrails (ICU 6/14, 42.9%; GW 0/12), and doorknobs (GW 1/12, 8.3%). Sporadic positive results were obtained from sleeve cuffs and gloves of medical staff. These results suggest that medical staff should perform hand hygiene practices immediately after patient contact.

Because patient masks contained exhaled droplets and oral secretions, the rate of positivity for those masks was also high (Tables 1, 2). We recommend adequately disinfecting masks before discarding them.

We further assessed the risk for aerosol transmission of SARS-CoV-2. First, we collected air in the isolation ward of the ICU (12 air supplies and 16 air discharges per hour) and GW (8 air supplies and 12 air discharges per hour) and obtained positive test results for 35% (14 samples positive/40 samples tested) of ICU samples and 12.5% (2/16) of GW samples. Air outlet swab samples also yielded positive test results, with positive rates of 66.7% (8/12) for ICUs and 8.3% (1/12) for GWs. These results confirm that SARS-CoV-2 aerosol exposure poses risks.

**Figure 2:**
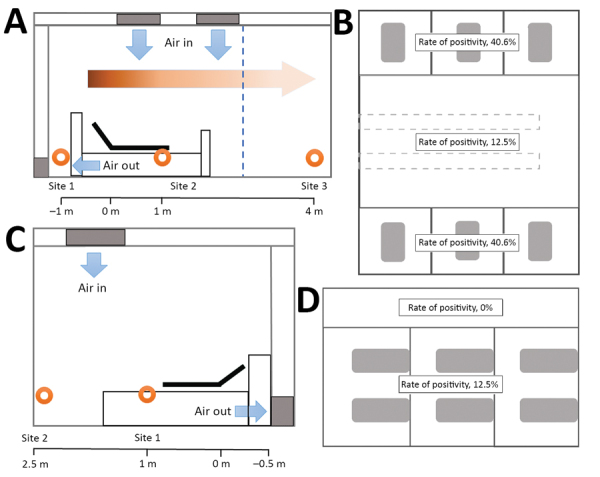
Spatial distribution of severe acute respiratory syndrome coronavirus 2 aerosols in isolation wards of the intensive care unit (ICU) and the general ward at Huoshenshan Hospital, Wuhan, China. A) The air sampling sites in the ICU were distributed in different regions: near the air outlet (site 1), near the patients (site 2), and around the doctors’ office area (site 3). Orange circles represent sampling sites; blue arrows represent direction of the fresh air flow; and the graded orange arrow and scale bar indicate the horizontal distance from the patient’s head. B) In terms of viral aerosol distribution, the space in the ICU was divided into 2 parts: a high-risk area with a 40.6% rate of virus positivity and a low-risk area with a 12.5% rate of virus positivity. C) The air sampling sites in the general ward were distributed in different regions around the patient (site 1), under the air inlet (site 2), and in the patient corridor. D) In terms of the viral aerosol distribution, the space in the general ward was divided into 2 parts: a high-risk area with a 12.5% rate of virus positivity and a low-risk area with a 0% rate of virus positivity.

Furthermore, we found that rates of positivity differed by air sampling site, which reflects the distribution of virus-laden aerosols in the wards (Figure 2, panel A). Sampling sites were located near the air outlets (site 1), in patients’ rooms (site 2), and (site 3). SARS-CoV-2 aerosol was detected at all 3 sampling sites; rates of positivity were 35.7% (5/14) near air outlets, 44.4% (8/18) in patients’ rooms, and 12.5% (1/8) in the doctors’ office area. These findings indicate that virus-laden aerosols were mainly concentrated near and downstream from the patients. However, exposure risk was also present in the upstream area; on the basis of the positive detection result from site 3, the maximum transmission distance of SARS-CoV-2 aerosol might be 4 m. According to the aerosol monitoring results, we divided ICU workplaces into high-risk and low-risk areas (Figure 2, panel B). The high-risk area was the patient care and treatment area, where rate of positivity was 40.6% (13/32). The low-risk area was the doctors’ office area, where rate of positivity was 12.5% (1/8).

In the GW, site 1 was located near the patients (Figure 2, panel C). Site 2 was located ≈2.5 m upstream of the air flow relative to the heads of patients. We also sampled the indoor air of the patient corridor. Only air samples from site 1 tested positive (18.2%, 2/11). The workplaces in the GW were also divided into 2 areas: a high-risk area inside the patient wards (rate of positivity 12.5, 2/16) and a low-risk area outside the wards (rate of positivity 0) (Figure 2, panel D).

## Conclusions

This study led to 3 conclusions. First, SARS-CoV-2 was widely distributed in the air and on object surfaces in both the ICU and GW, implying a potentially high infection risk for medical staff and other close contacts. Second, the environmental contamination was greater in the ICU than in the GW; thus, stricter protective measures should be taken by medical staff working in the ICU. Third, the SARS-CoV-2 aerosol distribution characteristics in the ICU indicate that the transmission distance of SARS-CoV-2 might be 4 m.

As of March 30, no staff members at Huoshenshan Hospital had been infected with SARS-CoV-2, indicating that appropriate precautions could effectively prevent infection. In addition, our findings suggest that home isolation of persons with suspected COVID-19 might not be a good control strategy. Family members usually do not have personal protective equipment and lack professional training, which easily leads to familial cluster infections (*6*). During the outbreak, the government of China strove to the fullest extent possible to isolate all patients with suspected COVID-19 by actions such as constructing mobile cabin hospitals in Wuhan (*7*), which ensured that all patients with suspected disease were cared for by professional medical staff and that virus transmission was effectively cut off. As of the end of March, the SARS-COV-2 epidemic in China had been well controlled.

Our study has 2 limitations. First, the results of the nucleic acid test do not indicate the amount of viable virus. Second, for the unknown minimal infectious dose, the aerosol transmission distance cannot be strictly determined.

Overall, we found that the air and object surfaces in COVID-19 wards were widely contaminated by SARS-CoV-2. These findings can be used to improve safety practices.

Appendix Supplementary methods for study of aerosol and surface distribution of severe acute respiratory syndrome coronavirus 2 in hospital wards, Wuhan, China, 2020.
