# Supplementary material for: Aerosol and Surface Distribution of Severe Acute Respiratory Syndrome Coronavirus 2 in Hospital Wards, Wuhan, China, 2020
Source: Emerg Infect Dis. 2020 Jul;26(7):1586–91. doi: 10.3201/eid2607.200885 (PMC7323510; doi:10.3201/eid2607.200885)
Supplement: Appendix — Supplementary methods for study of aerosol and surface distribution of severe acute respiratory syndrome coronavirus 2 in hospital wards, Wuhan, China, 2020. [file 20-0885-Techapp-s1.pdf]

Article DOI: https://doi.org/10.3201/eid2607.200885

# Aerosol and Surface Distribution of Severe Acute Respiratory Syndrome Coronavirus 2 in Hospital Wards, Wuhan, China, 2020

## Appendix

## Supplementary Methods

### 1. Collection of surface environmental samples

The sampling period was from February 19 to March 2, 2020 when 15 severe patients were treated in the ICU and 24 mild patients (2 in each isolation ward) were treated in the general ward.

Both the ICU and the general ward were cleaned twice daily, at ≈7:00 a.m. and 17:00 p.m. The cleaning routine included sweeping floor, wiping tables (with 1000 mg/L chlorine-containing disinfectant) and clearing rubbishes. The sampling monitoring was performed at 11:30 a.m., approximately four hours after the cleaning.

Sterile synthetic fiber swabs with plastic shafts were used to collect the surface environmental samples. Swabs were premoistened with viral transport media and wiped over the object surfaces for a few seconds. Then, Swabs were placed immediately into sterile tubes containing 2–3 mL of viral transport media. Each Swab was collected independently to avoid cross contamination. All samples were stored at 2–8°C and shipped to the testing laboratory within 4 h by ice pack to test for SARS-CoV-2. The results of SARS-CoV-2 test were available on the same day.

## 2. Quantitative real-time PCR assays

RNA was extracted using the LabServ® Prefilled Viral Total NA Kit-Flex and KingFisher Flex System (Thermo Fisher Scientific Inc., Waltham, USA) according the manufacturer's protocol. Quantitative real-time PCR (Q-RT-PCR) assays of SARS-CoV-2 open reading frame 1ab (ORF1ab) and nucleocapsid protein (NP) gene fragments were performed using the Novel Coronavirus (2019-nCoV) Nucleic Acid Diagnostic Kits (PCR-Fluorescence Probing) (Sansure Biotech Inc., Hunan, China) and CFX96 Touch Real-Time PCR Detection System (Bio-Rad Laboratories, Inc., California, USA). The diagnostic kit has passed the European Union CE-IVD certification and obtained the license of China Food and Drug Administration (National medical device registration certificate No. 20203400064). The limit of detection of this diagnostic kit is 200 copies/mL. Conditions for amplifications were 50°C for 30 min, 95°C for 1 min, followed by 45 cycles of 95°C for 15 s and 60°C for 30 s. Every assay contained a positive control and a negative control. Only both two controls produced the expected results, the data of samples were further analyzed. Otherwise, the data was invalid, and the experiment should be repeated. A sample was considered positive if either of the two targets (ORF1ab, NP) had an apparent logarithmic phase in the amplification curve and a cycle threshold value (Ct value) <40. In contrast, a sample was considered negative if both two targets had no apparent logarithmic phase or Ct value ≥40 or undetermined. In this study, intense positive indicated a positive result for both ORF1ab gene and N gene of SARS-CoV-2, while weak positive indicated a positive result for only one of the genes.
